# Abstract of the Proceedings of the Buffalo Medical Association

**Published:** 1864-05

**Authors:** J. F. Miner

**Affiliations:** Secretary


### ABSTRACT OF THE PROCEEDINGS OF THE BUFFALO MEDICAL ASSOCIATION.

Annual Meeting, April 5, 1864.

Minutes of the last meeting read and accepted. After the report of the Tteasurer for the past year and the transaction of miscellaneous business, there was made the following election of Officers for the ensuing year:

For President,......................John B. Samo, M. D.
" Vice President,....................Wm. Ring, M. D.
" Secretary,........................Joseph A. Peters, M. D.
" Treasuret,........................T. T. Lockwood, M. D.
" Librarian,........................T. M. Johnson, M. D.

Dr. John S. Trowbridge was proposed for membership.

Dr. Ayer was elected a member upon compliance with the by-laws.

J. F. Miner, Secretary.

